# Mortality Table

**Published:** 1879-09

**Authors:** 


### MORTALITY TABLE.

Condensed from National Board of Health Bulletin, for the four weeks, ending August 2d, 1879.

| CITIES. | Estimated Population. | Deaths. | Death rate per 1000. |
|---|---|---|---|
| BALTIMORE, - - - - - | 400,000 | 733 | 23.55 |
| BOSTON, - - - - - | 365,000 | 601 | 21.40 |
| **BUFFALO**, - - - - | **170,000** | †—— | —— |
| CHICAGO, - - - - - | 460,000 | 960 | 27.13 |
| CLEVELAND, - - - - - | 160,000 | 338 | 27.43 |
| LOUISVILLE, - - - - - | 175,000 | 228 | 16.94 |
| NEW YORK, - - - - - | 1,097,000 | 2779 | 32.90 |
| PHILADELPHIA, - - - - | 901,000 | 1580 | 22.78 |
| ROCHESTER, - - - - - | 90,000 | *132 | 17.6 |
| ST. LOUIS, - - - - - | 500,000 | 692 | 17.99 |

* For the month of July, 1879. † No report furnished by Nat. B'd of Health.

